# Genetic Variants of the NKG2C/HLA-E Receptor–Ligand Axis Are Determinants of Progression-Free Survival and Therapy Outcome in Aggressive B-Cell Lymphoma

**DOI:** 10.3390/cancers12113429

**Published:** 2020-11-18

**Authors:** Bettina Wagner, Ulrich Dührsen, Andreas Hüttmann, Holger Nückel, Rafael Tomoya Michita, Hana Rohn, Sabine Schramm, Peter A. Horn, Vera Rebmann

**Affiliations:** 1 Institute for Transfusion Medicine, University Hospital Essen, University Duisburg-Essen, 45147 Essen, Germany; Bettina.Wagner@stud.uni-due.de (B.W.); sabine.schramm3@uk-essen.de (S.S.); Peter.Horn@uk-essen.de (P.A.H.); 2 Department of Hematology, West German Cancer Center, University Hospital Essen, University of Duisburg-Essen, 45147 Essen, Germany; Ulrich.Duehrsen@uk-essen.de (U.D.); andreas.huettmann@uk-essen.de (A.H.); 3 Medical Practice for Hematology, Oncology, Hemostaseology and Palliative Care, 44787 Bochum, Germany; Holger.Nueckel@uk-essen.de; 4 Department of Genetics, Institute of Biosciences, Universidade Federal do Rio Grande do Sul (UFRGS), Bento Gonçalves Avenue 9500, Campus do Vale, Porto Alegre, RS CEP 91501970, Brazil; rafael.michita@rede.ulbra.br; 5 Department of Infectious Diseases, West German Centre for Infectious Diseases (WZI), University Hospital Essen, University Duisburg-Essen, 45147 Essen, Germany; Hana.Rohn@uk-essen.de

**Keywords:** HLA-E, NKG2C, NHL, aggressive lymphoma

## Abstract

**Simple Summary:**

NKG2C and its ligand HLA-E represent key molecules for NK cell-mediated immune responsiveness. However, the impact of genetic variants in *NKG2C* and *HLA-E* on clinical outcomes of aggressive B-cell non-Hodgkin lymphoma patients (B-NHL) has not been clarified. In this study, we analyzed the distribution of *NKG2C* deletion status and HLA-E variants in 441 patients and 192 healthy individuals. Homozygous deletion of *NKG2C* (*NKG2C*^−/−^) was more often found in high-risk patients compared to patients with a lower risk and consequently was associated with reduced 2-year progression-free survival. The HLA-E*01:01 allele frequency was increased in B-NHL patients and was strongly related with complete remission. Our results show that absence of *NKG2C* and *HLA-E* allelic variations is predictive for B-NHL outcome; while carriers of HLA-E*01:01 are characterized by high, complete remission rates, *NKG2C*^−/−^ was rare, but associated with poorer outcome. Prospective validation of our results identifies patients that may benefit from risk-adapted therapy.

**Abstract:**

Aggressive B-cell lymphomas account for the majority of non-Hodgkin lymphomas (B-NHL). NK cells govern the responses to anti-CD20 monoclonal antibodies and have emerged as attractive targets for immunotherapy in subtypes of B-NHL. NKG2C and its cognate ligand HLA-E represent key molecules for fine-tuning of NK cell-mediated immune responses. Here, we investigated the impact of genetic variants of *NKG2C* and *HLA-E* on clinical outcomes of 441 B-NHL patients. Homozygous deletion of *NKG2C* (*NKG2C^−/−^*) was three-fold increased in patients compared to 192 healthy controls. Among studied patients, *NKG2C^−/−^* was more abundant in International Prognostic Index (IPI) high-risk patients compared to patients with a lower IPI (*p* = 0.013). Strikingly, *NKG2C^−/−^* was associated with a significantly reduced 2-year PFS (progression-free survival) (*p* = 0.0062) and represented an independent risk factor for 2-year PFS in multivariate analysis (*p* = 0.005). For HLA-E, the cognate ligand of NKG2C, the HLA-E*01:01 allele frequency was increased in B-NHL patients compared to controls (*p* = 0.033) and was associated with complete remission in univariate (*p* = 0.034) and multivariate (*p* = 0.018) analysis. Our data suggest that *NKG2C* and *HLA-E* genotyping is a promising tool for both defining risk groups of aggressive B-NHL and predicting response to immune therapeutic approaches.

## 1. Introduction

Aggressive B-cell non-Hodgkin lymphoma (B-NHL) represents malignant tumors of the immune system [1]. First-line treatment consists of the CHOP regimen (cyclophosphamide, doxorubicin, vincristine, and prednisone). The combined treatment with the anti-CD20 antibody rituximab (R-CHOP) has improved the therapy outcome of CD20 positive B-NHL patients with long-term remission rates of 60% to 70%. Consequently, up to 40% of patients experience treatment failure or disease relapse [2]. Therefore, surrogate markers identifying subgroups of patients with high-risk of treatment failure or early disease progression are of paramount importance for precision medicine approaches. The established risk model for B-NHL is based on clinical parameters summarized in the International Prognostic Index (IPI) [3]. However, molecular markers of the primary tumor or of immune effectors are currently not implemented in routine clinical care of B-NHL patients.

In recent years, increasing evidence suggests that NK cells as effectors of the innate immune system are critically involved in surveillance of B-NHL [4]. Particularly, NK cells have been discussed to support the therapeutic effect of CD20-targeting treatment approaches in lymphoma patients by strong Fc receptor-mediated NK cell activation, boosting the antibody-dependent cellular cytotoxicity (ADCC) [4,5]. Immune reactivity of NK cells is largely governed by the inhibitory lectin like receptors NK group 2 member A (NKG2A), and the counterbalancing activating NKG2C receptor, both of which are expressed as heterodimers with the invariant CD94 on the cell surface [6,7,8,9,10]. Expression of NKG2C is a feature of so-called adaptive NK cells operative in inflammation and infections [11,12,13]. CD94-NKG2 receptors recognize the non-classical HLA-E molecule. While HLA-E is widely expressed at low levels under physiological conditions [14,15], it can be up-regulated during pathological processes, including cancer [16,17,18]. HLA-E expression is a critical component for modulating immune responses as it can interact with the activating NKG2C as well as the inhibitory NKG2A receptor.

Genetic variants in *NKG2C* and *HLA-E* have been identified and functionally characterized. It has been described that heterozygous or homozygous deletion of a 16-kb section within the *NKG2C* gene correlates with decrease or absence of NKG2C surface expression, respectively [19,20]. Thus, the copy number of variations affects the adaptive response of NKG2C positive NK cells [12,21,22]. For HLA-E, two main genetic variants, HLA-E*01:01 and HLA-E*01:03, are equally distributed in populations of different origins [23,24]. However, the HLA-E*01:03 encoded protein shows a higher peptide-binding affinity and higher surface expression compared to the HLA-E*01:01 encoded protein [24,25,26]. Currently, the role of genetic variations in *NKG2C* and *HLA-E* in balancing anti-tumor immune response or disease outcome in hematological malignancies has not been investigated.

As the NKG2C/HLA-E axis is a central element for fine-tuning the immune responsiveness of NK cells, we here addressed the question if genetic variations in the encoding genes were relevant for clinical outcome and therapy response of B-NHL. We determined allele and genotype frequencies of the *NKG2C* deletion and *HLA-E* variants in CD20 positive B-NHL patients (*n* = 441) and healthy controls (*n* = 192) and related the results of the patients with clinical characteristics as well as disease outcome.

## 2. Results

### 2.1. Homozygous Absence of NKG2C Is More Frequent in International Prognostic Index (IPI) High-Risk Than in IPI Non-High-Risk Patients

While *NKG2A* and *NKG2C* are both located on chromosome 12, only the *NKG2C* locus is deleted in 4–14% of individuals depending on the population analyzed. Here, *NKG2C* genotype distribution was determined in B-NHL patients (*n* = 441) and controls (*n* = 189), respectively. Neither *NKG2C* deletion nor genotype frequencies of copy number variations were different between B-NHL patients and healthy controls (Table 1). Although homozygous *NKG2C* deletion (*NKG2C^−/−^*) frequency was two-fold higher in B-NHL patients (4.5%) than in healthy controls (1.6%), this finding, however, did not reach statistical significance.

**Figure 1:**
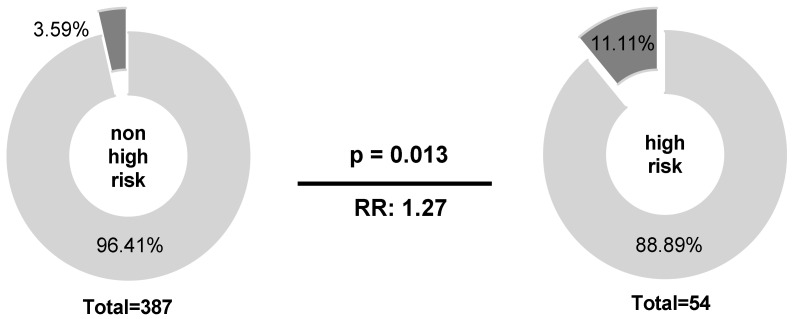
Correlation of IPI score and the *NKG2C^−/−^* status. Higher frequencies of the *NKG2C^−/−^* could be observed for patients in the IPI high-risk group (IPI 4–5) compared to those in the non-high-risk group (IPI 0–3; low to high-intermediate risk). *NKG2C^−/−^* (dark grey), *NKG2C*^+/−^ or *NKG2C*^+/+^ (light grey). Clinical data were not available for all patients.

Among B-NHL patients, IPI score was the only clinical parameter associated with *NKG2C^−/−^* status (*p* = 0.013; *n* = 441). Here, *NKG2C^−/−^* status was 3-fold increased in the IPI high-risk group compared to patients with a lower IPI score (*p* = 0.013; RR: 1.27; 95% CI: 1.032 to 1.844; Figure 1).

### 2.2. Homozygous Absence of NKG2C Is Associated With Reduced Two-Year PFS

**Figure 2:**
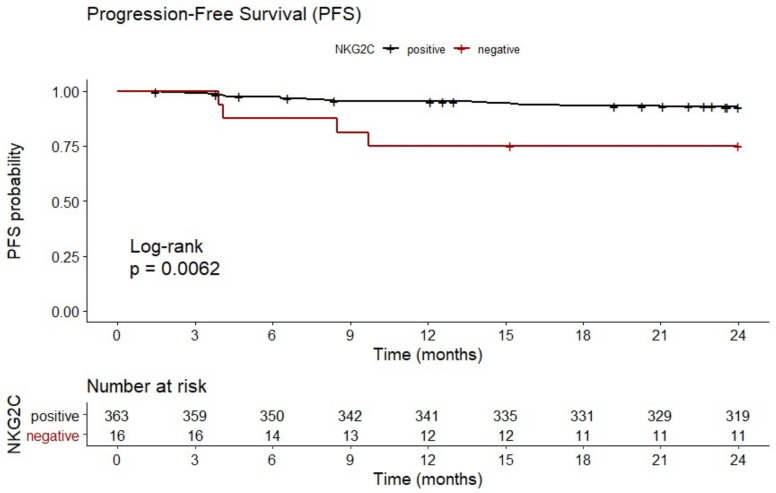
Reduced 2-year progression-free survival (PFS) in patients with *NKG2C^−/−^*. B-NHL patients with homozygous deletion of *NKG2C* showed a reduced 2-year PFS compared to *NKG2C* positive patients. *NKG2C^−/−^* (negative, red); *NKG2C*^+/−^ or *NKG2C*^+/+^ (positive, black). Data were not available for the total number of patients due to loss of follow up.

In line with enhanced IPI risk scores, patients with *NKG2C^−/−^* experienced a reduced progression-free survival (PFS) (19.1 ± 7.9 months) compared to *NKG2C* positive patients with *NKG2C*^+/−^ or *NKG2C*^+/+^ (22.5 ± 4.9 months; *p* = 0.0062; HR: 3.88; 95% CI: 0.60 to 25.09; *n* = 379; Figure 2).

**Figure 3:**
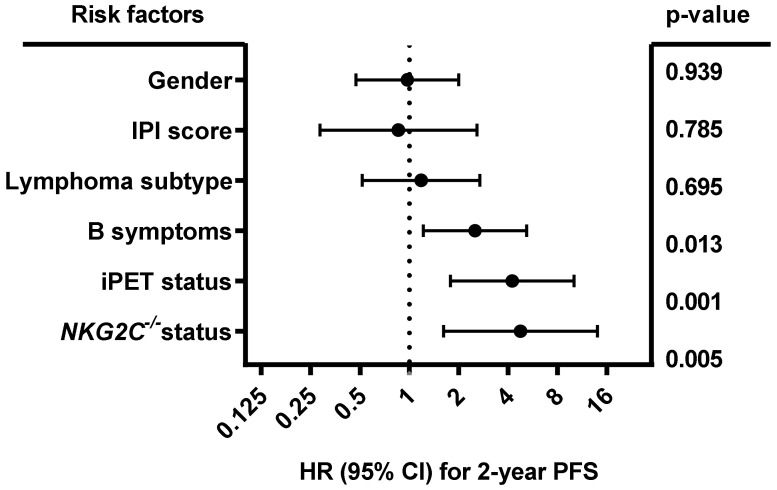
Forest plot of risk factors for 2-year PFS. The forest plot visualizes the multivariate analysis of the following parameters: Gender (male vs. female), IPI (0–3 vs. 4–5), lymphoma subtype (DLBCL vs. other), B symptoms (A vs. B), iPET status (favorable vs. unfavorable iPET), and *NKG2C^−/−^* status (*NKG2C^−/−^* vs. *NKG2C*^+/−^ or *NKG2C*^+/+^). The analysis showed that besides B symptoms and iPET status, the *NKG2C* status is an independent prognostic marker of 2-year PFS in B-NHL patients. Due to loss of follow up, data were available for 379 out of 441 patients. CI (confidence interval), HR (hazard ratio), PFS (progression-free survival at end of treatment).

As shown in Figure 3, multivariate analysis including gender (male vs. female), IPI (0–3 vs. 4–5), lymphoma subtype (diffuse large B-cell lymphoma (DLBCL) vs. other), B symptoms (A vs. B), iPET status (favorable iPET vs. unfavorable iPET) and *NKG2C^−/−^* status (*NKG2C^−/−^* vs. *NKG2C*^+/−^ or *NKG2C*^+/+^) showed that besides B symptoms (*p* = 0.013; HR:2.51; 95% CI: 1.218 to 5.173) and iPET (*p* = 0.001; HR: 4.242; 95% CI: 1.781 to 10.102), the *NKG2C^−/−^* status was an independent predictive factor for 2-year PFS in B-NHL patients (*p* = 0.005; HR: 4.758; 95% CI: 1.617 to 13.999; *n* = 379). No association of *NKG2C* absence with the overall survival was found in univariate analysis (data not shown).

### 2.3. HLA-E*01:01 Allele Frequencies Are Increased in B-NHL Patients

To analyze the impact of HLA-E as cognate ligand of NKG2C, *HLA-E* genotyping was performed in patients (*n* = 441) and controls (*n* = 192), respectively (Table 2). HLA-E*01:01 allele frequencies were increased in B-NHL patients compared to healthy controls (57.3% vs. 50.8%; RR: 1.083, 95% CI: 1.006 to 1.168; OR: 1.298; *p* = 0.0332), whereas the *HLA-E* genotypes were not found to be different between patients and controls. Furthermore, *HLA-E* allele or *HLA-E* genotype frequencies were not associated with clinical or prognostic parameters (data not shown).

### 2.4. HLA-E*01:01 Is Associated with CR in B-NHL Patients

**Figure 4:**
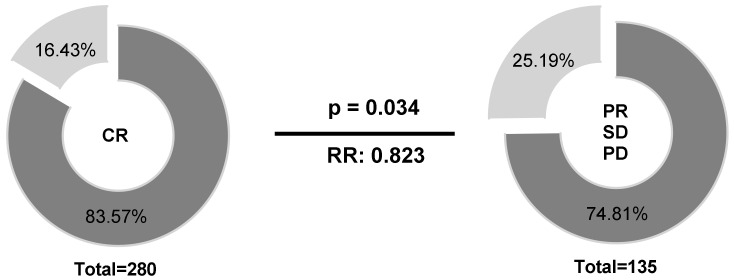
Association of the HLA-E*01:01 allele carrier status according to therapy response. Higher frequencies of HLA-E*01:01 allele carriers could be observed in patients with CR compared to patients with limited therapy response. HLA-E*01:01/01:01 or HLA-E*01:01/01:03, dark grey; HLA-E*01:03/01:03; light grey; PD = progressive disease, SD = stable disease, PR = partial remission, CR = complete remission; Clinical data were available for 415 out of 441 patients.

Regarding therapy response at the end of treatment, patients (*n* = 441) were stratified into two groups according to their HLA-E*01:01 allele carrier status (HLA-E*01:01/*01:01 or HLA-E*01:01/01:03 vs. HLA-E*01:03/01:03). Univariate analysis (Figure 4) revealed higher frequencies of HLA-E*01:01 allele carriers (83.57% vs. 74.81%) in patients with complete remission (CR) compared to patients with limited therapy response (*p* = 0.034; RR: 0.823; 95% CI: 0.66 to 0.987).

**Figure 5:**
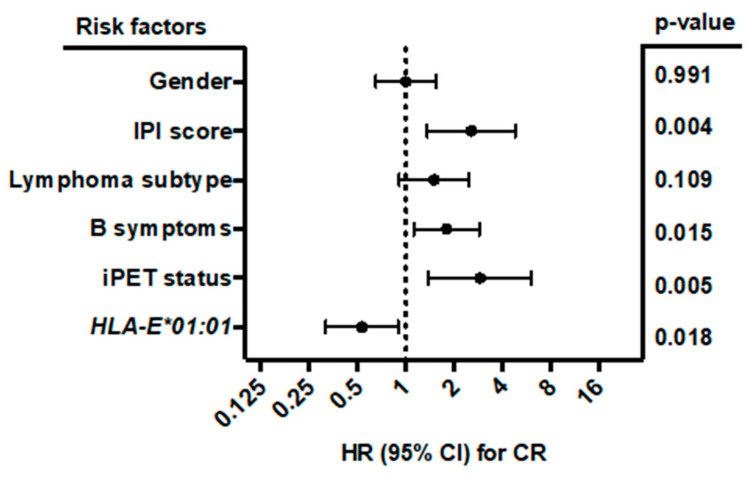
Forest plot of risk factors for CR. The forest plot visualizes the multivariate analysis (binomial logistic regression) of the following parameters: gender (male vs. female), IPI score (0–3 vs. 4–5), lymphoma subtype (DLBCL vs. other), B symptoms (A vs. B), iPET status (favorable vs. unfavorable iPET), and HLA-E*01:01 allele carrier status (HLA-E*01:01/*01:01 or HLA-E*01:01/01:03, vs. HLA-E*01:03/01:03). All clinical data were available for 414 out of 441 patients. The analysis showed that besides B symptoms, IPI score, and iPET status, the HLA-E*01:01 allele carrier status was an independent prognostic risk factor for the prediction of CR. CI = confidence interval, HR = hazard ratio, CR = complete remission.

Multivariate analysis (Figure 5) including gender (male vs. female), B symptoms (A vs. B), IPI score (0–3 vs. 4–5), lymphoma subtype (DLBCL vs. other), iPET status (favorable iPET vs. unfavorable iPET), and HLA-E*01:01 allele carrier status (HLA-E*01:01/*01:01 or HLA-E*01:01/01:03 vs. HLA-E*01:03/01:03) showed that the HLA-E*01:01 allele carrier status was an independent prognostic factor correlating with complete remission in B-NHL patients at the end of treatment (*p* = 0.018; HR: 0.534; 95% CI: 0.318 to 0.898; *n* = 414). Both homozygous and heterozygous HLA-E*01:01 allele carriers had the same clinical correlation. By contrast, B symptoms (*p* = 0.015; HR: 1.796; 95% CI: 1.12 to 2.88), higher IPI score (*p* = 0.004; HR: 2.561; 95% CI: 1.361 to 4.819) and unfavorable iPET status (*p* = 0.005; HR: 2.902; 1.386 to 6.076) were significantly associated with less favorable treatment outcome.

## 3. Discussion

The interplay of NKG2C/A receptors with their ligand HLA-E is thought to play a crucial role in the fine-tuning of immune responses [9,10]. Genetic variations of these molecules are known to affect the expression or function of encoded proteins [12,19,24] and may have an impact on NKG2C/A-HLA-E-mediated immune reactions.

In our study, we focused on the diagnostic and prognostic potential of *NKG2C* gene deletion and *HLA-E* allelic variations in patients with B-NHL. We here demonstrate for the first time that the homozygous *NKG2C* deletion status (i) is increased in high risk B-NHL patients; (ii) is associated with a reduced 2-year PFS in univariate analysis, and (iii) represents an independent prognostic factor for 2-year PFS in multivariate analysis. For HLA-E, the corresponding ligand of *NKG2C*, frequencies of the HLA-E*01:01 allele are increased in B-NHL patients, and the HLA-E*01:01 status is related with complete remission in both univariate and multivariate analysis.

The low frequency of homozygous *NKG2C* deletion (4.5%) in B-NHL patients and healthy controls (1.6%) in this study are comparable to other population studies including Japanese, Dutch [20], Mexican [27], German [12] and Spanish investigations [28]. So far, the impact of the *NKG2C* gene deletion has been shown to be associated with the susceptibility to psoriasis [11] and viral infections [12,29,30], but this has not yet been described in the context of hematological malignancies. In our study, the homozygous *NKG2C* deletion was found at a higher frequency in B-NHL patients compared to healthy controls without reaching statistical significance. By contrast, *NKG2C* deletion was detected more often in high-risk than low-risk patients (11.11% vs. 3.59%). Here, the absence of NKG2C may favor the risk of progression in B-NHL patients as HLA-E bearing tumor cells are not being recognized by NK cells, while the probability of an inhibition by the NKG2A receptor may rise. Noticeably, parameters being incorporated in the IPI score, including age, performance status (ECOG), Ann Arbor stage, extra nodal manifestation, and elevated LDH are independent from the homozygous *NKG2C* deletion status (data not shown).

The successful implementation of the IPI score as a model to identify high-risk subgroups of B-NHL patients with unfavorable PFS or OS (overall survival) in clinical trials enrolling patients from different health care centers is controversially discussed [3,31,32,33]. Based on our results, homozygous *NKG2C* deletion could serve as a molecular marker, which may be considered for risk stratification of aggressive B-NHL, although this is restricted to a minor fraction of patients. However, patients with a homozygous *NKG2C* deletion also suffer from a reduced 2-year PFS at end of treatment, supporting the notion that the *NKG2C* genotype could be clinically relevant. Multivariate analysis revealed that homozygous *NKG2C* deletion was an independent prognostic risk factor for the prediction of 2-year PFS, whereas IPI was not. A recent report also emphasized that distinct molecular alterations, structural variants, and recurrent mutations, were predictive of disease outcome independent of the clinical IPI score in DLBCL subtypes [34] and thereby were a meaningful complementary parameter for risk stratification.

Accumulating evidence suggests that NK cells contribute to successful elimination of malignant B-cells during rituximab therapy [35,36]. Of note, all patients in our cohort were treated with the antibody rituximab. So-called adaptive NK cells expressing high level of the activating receptor NKG2C [21,22] and CD57 NK cells display enhanced capacity to produce IFN-γ in response to antibody-coated target cells, suggesting that NKG2C positive NK cells are better equipped for cytokine production upon rituximab treatment. Although the functional consequences of NKG2C absence in B-NHL patients have to be investigated in further studies, it is tempting to speculate that absence of the NKG2C positive cells may impair NK cell-mediated support of rituximab treatment, especially since previous studies demonstrated that low copy numbers of *NKG2C* were associated with impaired NK cell activation or proliferation [12,13]. In the absence of NKG2C-mediated activation, NK cells may be more prone to inhibition by NKG2A/HLA-E interactions, leading to early progression. Thus, NKG2C deficient B-NHL patients may benefit from NKG2A blocking. For these patients, the monoclonal antibody monalizumab, which blocks NKG2A-mediated signaling, may serve as an additional immune therapeutic option [37].

For the cognate ligand of NKG2C, HLA-E, two main alleles exist that are equally present in all populations analyzed [23]. HLA-E*01:01 is linked to comparatively low HLA-E surface expression, whereas substantially higher HLA-E expression is found in the presence of at least one HLA-E*01:03 allele in healthy individuals [6,24,25] as well as in hematological disorders such as acute leukemia [9] or chronic lymphocytic leukemia [10]. Several studies reported a higher prevalence of the HLA-E*01:03 allele or the HLA-E*01:03/01:03 genotype in infectious diseases [38] and several cancer types including nasopharyngeal carcinoma, serous ovarian cancer, and acute leukemia [9,39,40]. Interestingly, the HLA-E*01:01 allele seems to be underrepresented in classical Hodgkin lymphomas, especially among Epstein Barr virus (EBV)-positive cases compared to healthy controls [41]. We observed a slightly higher prevalence of the HLA-E*01:01 genotype in aggressive B-NHL. Importantly, increased frequency of HLA-E*01:01 allele carriers was related with complete remission after the end of treatment in univariate and multivariate analysis. Here it is likely that the low surface expression of the HLA-E*01:01 allele might attenuate the inhibition of NK cells via NKG2A/HLA-E interactions, which in turn supports the antibody-dependent cellular cytotoxicity of NK cells during rituximab treatment favoring a complete remission. Nevertheless, this observation has to be confirmed in independent investigations. Notably, genome wide analyses show that classical HLA is associated with lymphoma and is suggested to be an independent factor for disease risk [42].

## 4. Materials and Methods

### 4.1. Patients and Controls

The multicenter study PETAL (Positron Emission Tomography-Guided Therapy of Aggressive Non-Hodgkin Lymphomas) randomized controlled trial (EudraCT 2006-001641-33, ClinicalTrials.gov NCT00554164) enrolled patients with newly diagnosed aggressive B-cell or T-cell lymphomas (age 18–80 years) and an Eastern Co-operative Oncology Group (ECOG) performance status ≤ 3 [43]. All subjects gave their informed consent for inclusion before they participated in the study. The study was conducted in accordance with the Declaration of Helsinki, and the protocol was approved by the Ethics Committee of BfArM, Germany (61-3910-4032976; date of approval: 11 June 2007) and ethics committee of all participating centers coordinated by the Ethics Committee of the Medical Faculty of the University of Duisburg-Essen (07-3366; date of approval: 25 July 2007).

Here, we focused on a subgroup of this study cohort including patients with CD20 positive aggressive B-cell lymphoma (B-NHL) and a positive baseline positron emission tomography (PET, *n* = 441) receiving two cycles of R-CHOP. After the pretreatment, patients were randomized and allocated according to the interim PET (iPET) scan result in groups, with favorable iPET (*n* = 396) being treated either with four cycles of R-CHOP or with the same the number of cycles but with two additional doses of rituximab, and with unfavorable iPET (*n* = 42) receiving either six additional cycles of R-CHOP or six blocks of an intensive protocol originally designed for Burkitt’s lymphoma [44]. PET scans were evaluated using the ΔSUVmax method [43,45]. The demographic profile of the B-NHL patients included in this sub-study is summarized in Table 3. Seventy-seven male and 115 female healthy blood donors (*n* = 192) with a mean age of 51.31 ± 9.95 years were used as controls.

### 4.2. NKG2C and HLA-E Genotyping

For patients and controls, genotyping of the *NKG2C* deletion or *HLA-E* was performed as previously described [20,46]. *NKG2C* and *HLA-E* genotype distributions in patients and controls did not significantly deviate from the Hardy-Weinberg equilibrium.

### 4.3. Statistical Analysis

Metric data are given as mean ± standard deviation. Chi-square tests were used to analyze categorical data. Binomial logistic regression was used to identify clinical parameters influencing the therapy response after ending of treatment. Progression-free survival (PFS) and overall survival (OS), respectively, were defined as the time from the date of iPET to disease progression or death from any cause. Probabilities of OS and PFS were analyzed using the Kaplan–Meier method in combination with the log-rank test implemented in the R package survminer (version 0.4.0; https://CRAN.R-project.or/package=survminer). Multivariate Cox regression according to proportional hazards assumption was used to identify prognostic factors for PFS or OS. *p* values < 0.05 were considered statistically significant. Remaining statistical analyses were performed using either SPSS 23.0 (SPSS Inc, Chicago, IL, USA), GraphPad Prism 8.3.1 (GraphPad Software, San Diego, CA, USA) or GENEPOP 4.7 (https://genepop.curtin.edu.au/).

## 5. Conclusions

In conclusion, our results provide substantial evidence that absence of *NKG2C* and *HLA-E* allelic variations are predictive of outcomes in B-NHL; while allele carriers of HLA-E*01:01 are characterized by high complete remission rates, *NKG2C* absence is rare but significantly associated with poorer outcomes. Prospective validation of our results is warranted to identify patient subgroups, which may provide benefits from risk-adapted therapy.

## Figures and Tables

**Table 1:** Distribution of *NKG2C* allelic and genotype frequencies of B-cell non-Hodgkin lymphoma (B-NHL) patients and healthy controls. The *NKG2C* homozygous deletion allele frequencies and genotype frequencies did not differ among B-NHL patients and healthy controls.

Variable	B-NHL Patients *n* = 441 *n* (%)	Controls *n* = 189 *n* (%)	*p*-Value
**Alleles (2n)**			
*NKG2C^−^*	173 (19.6)	77 (20.4)	0.7579
*NKG2C^+^*	709 (80.4)	301 (79.6)	
**Genotypes**			
*NKG2C^−/−^*	20 (4.5)	3 (1.6)	0.0706
*NKG2C^+/−^*	133 (30.2)	71 (37.6)	0.0686
*NKG2C^+/+^*	288 (65.3)	115 (60.8)	0.2853

**Table 2:** Distribution of *HLA-E* allelic and genotype frequencies of patients and controls. HLA-E*01:01 allele frequencies were increased in B-NHL patients compared to healthy controls. The genotype frequencies did not differ between patients and controls.

Variable	B-NHL Patients *n* = 441 *n* (%)	Controls *n* = 192 *n* (%)	*p*-Value
**Alleles (2n)**			
HLA-E*01:01	505 (57.3)	195 (50.8)	0.0332
HLA-E*01:03	377 (42.7)	189 (49.2)	
**Genotypes**			
HLA-E*01:01/*01:01	147 (33.3)	52 (27.1)	0.1195
HLA-E*01:01/*01:03	211 (47.8)	91 (47.4)	0.9170
HLA-E*01:03/*01:03	83 (18.8)	49 (25.5)	0.0565

**Table 3:** Demographic profile of B-NHL patients. iPET (interim positron emission tomography); International Prognostic Index (IPI) stratifying patients in four risk groups (0–1 factor positive = low risk, 2 factors positive = low-intermediate risk, 3 factors positive = high-intermediate risk, 4–5 factors positive = high risk); ECOG performance status (0–1, 2–3); B symptoms (A = no B symptoms; B ≥ 1 B symptom including fever > 38 °C, drenching night sweats, weight loss > 10 kg); diffuse large B-cell lymphoma (DLBCL); other included: follicular lymphoma (FL) with FL grade 1–2, grade 3 with areas of grades 1 or 2, grade 3; primary mediastinal B-cell lymphoma (PMBL); mantle cell lymphoma, unclassified B-cell lymphoma and mixed histologies; complete remission (CR), partial remission (PR), progressive disease (PD), stable disease (SD); progression-free survival (PFS); overall survival (OS); * data were not available for the total number of patients.

**Parameter**		***n***
		441
Age	≤60	145
	>60	296
Gender	female	213
	male	228
ECOG *	0–1	397
	2–3	40
B symptoms *	A	321
	B	119
Ann Arbor *	I–II	190
	III–IV	250
Extra nodal manifestation (>1) *	No	309
	Yes	131
Elevated LDH *	No	205
	Yes	235
IPI risk group *	Low risk	185
	Low-intermediate risk	103
	High-intermediate risk	99
	High risk	53
Lymphoma subtype	DLBCL	339
	other	102
iPET *	favorable	396
	unfavorable	42
Therapy response end-of-treatment *	CR	280
	PR, PD, SD	135
2-year PFS *	No event	349
	Event	30
2-year OS *	Alive	404
	Dead	30
